# A nationwide analysis of the outcomes in hospitalized patients with atrial fibrillation and temperature-related illnesses

**DOI:** 10.1016/j.clinsp.2023.100269

**Published:** 2023-08-07

**Authors:** Daniel Antwi-Amoabeng, Sunil Sathappan, Tarek R. Firzli, Bryce D. Beutler, Mark B. Ulanja, T. David Gbadebo

**Affiliations:** a Christus Ochsner St. Patrick Hospital, Lake Charles, USA; b University of Nevada, Reno School of Medicine, Reno, USA; c University of Southern California, Keck School of Medicine, Los Angeles, USA; d Emory Decatur Hospital, Cardiology Section, GA, USA

**Keywords:** Atrial fibrillation, Cold-related injury, Heat-related injury, Heatstroke, Frostbites, National inpatient sample

## Abstract

**Objectives:**

The authors evaluated mortality and indices of cost of care among inpatients with Atrial Fibrillation (AF) and a diagnosis of a Temperature-Related Illness (TRI). The authors also assessed trends in the prevalence of TRIs among AF hospitalizations.

**Methods:**

In this cross-sectional study, the authors used discharge data from the Nationwide Inpatient Sample (NIS) collected between January 2005 and September 2015 to identify patients with a diagnosis of AF and TRI. Outcomes of interest included in-hospital mortality, invasive mechanical ventilation, hospital length of stay, and cost of hospitalization.

**Results:**

A total of 37,933 encounters were included. The median age was 79 years. Males were slightly overrepresented relative to females (54.2% vs. 45.8%, respectively). Although Blacks were only 6.6% of the cohort, they represented 12.2% of the TRI cases. Compared to non-TRI-related hospitalizations, a diagnosis of a TRI was associated with an increased likelihood of invasive mechanical ventilation (16.5% vs. 4.1%, *p* < 0.001), longer length-of-stay (5 vs. 4 days, *p <* 0.001), higher cost of care (10,082 vs. 8,607, in US dollars *p <* 0.001), and increased mortality (18.6% vs. 5.1%, *p <* 0.001). Compared to non-TRI, cold-related illness portends higher odds of mortality 4.68, 95% Confidence Interval (4.35–5.04), *p <* 0.001, and heat-related illness was associated with less odds of mortality, but this was not statistically significant 0.77 (0.57–1.03), *p* = 0.88.

**Conclusion:**

The occurrence of TRI among hospitalized AF patients is small but there is an increasing trend in the prevalence, which more than doubled over the decade in this study. Individuals with AF who are admitted with a TRI face significantly poorer outcomes than those admitted without a TRI including higher mortality. Cold-related illness is associated with higher odds of mortality. Further research is required to elucidate the pathogenic mechanisms underlying these findings and identify strategies to prevent TRIs in AF patients.

## Highlights

• Temperature-Related Illness (TRI) describes physiologic aberrations that occur secondary to exposure to extreme cold or heat.
• Individuals with Atrial Fibrillation (AF) who present with TRI may be at increased risk of poor outcomes relative to patients without TRI.
• Our analysis shows that individuals with AF who develop a cold-related TRI face an increased risk of mortality as compared to those without TRI.

## Introduction

Temperature-Related Illness (TRI) represents a spectrum of injury or pathological manifestations from exposure to non-optimal environmental temperatures. The exact thresholds at which temperature-related illnesses occur vary, based on a variation in an individual's tolerance of weather extremes, comorbid medical conditions, prevailing weather conditions the time spent within such temperatures. Hypothermia-induced injury is likely to occur during prolonged periods below 0 °C and 4.4 °C with wind chill.^1^ While hyperthermia-related injury is likely to occur at 41.6 °C to 42 °C.^2^ These injuries can manifest in a variety of ways. Cold-weather events can trigger frostbite, chilblains, and hypothermia.^3^ Hot-weather events can induce hyperthermia, heat exhaustion, and heatstroke, among others.^4^ Both non-optimal cold and hot temperatures are responsible for an estimated 74 excess deaths per 100 000 persons based on a global study.^5^

TRIs have likewise been demonstrated to adversely influence cardiovascular outcomes and have been reported to contribute to an increased risk of cardiopulmonary hospitalization and death.^6^, ^7^, ^8^ There is a 5% increase in the relative risk of arrhythmia-related mortality for every 1 °C elevation in outdoor temperatures.^7^ On the other hand, there is a 16% increased odds of ventricular arrhythmias for each 1 °C decrease in the 24-hour average indoor temperature.^9^ Although non-optimal temperatures may influence the incidence of arrhythmias, it was not associated with increased rates of hospitalization from arrhythmias including atrial fibrillation.^10^ However, both adversely low and high temperatures are associated with Atrial Fibrillation (AF) occurrence and mortality to different degrees.^11^, ^12^, ^13^

This study aimed to assess trends in the prevalence of temperature-related illness among AF hospitalizations over a decade from the years 2005 to 2015, and to assess the influence of TRI on mortality, need for mechanical ventilation, length of stay, and cost of hospital stay. With the increasing occurrence of extreme weather events, understanding the relationship between AF outcomes and TRI as well as the regional variations if any may provide valuable information in the planning of public health education interventions such as weather advisory warnings.

## Materials and methods

### Summary of data source

The discharge data from the Nationwide Inpatient Sample (NIS prior to 2012 data) and the National Inpatient Sample (NIS beginning with 2012 data) Healthcare Cost and Utilization Project (HCUP), Agency for Healthcare Research and Quality (AHRQ) were used for this study.^14^ The NIS is the largest publicly available all-payer inpatient care database which provides estimates for nationwide and regional inpatient utilization, cost of care, and outcomes among other quality measures in the United States. It systematically samples discharge encounters from 20% of all hospitalizations nationwide. This study did not require approval from our Institutional Review Board (IRB) because it uses a limited dataset, which is publicly available.

### Study population and outcomes

Atrial fibrillation was identified as the primary diagnosis in hospitalizations from January 2005 to September 2015 using the International Classifications of Diseases, Ninth Revision, Clinical Modification (ICD-9-CM) diagnostic code, 427.31, which has been used in previous studies.^15^, ^16^, ^17^ Temperature-related illness was defined as the occurrence of either cold- or heat-related injuries using ICD-9 codes: Heat-related illness: 9920, 9921, 9922, 9923, 9924, 9925, 9927, 9928, 9929, E9000, E9001, E9009,^4^ and Cold-related illness: 9910, 9911, 9912, 9913, 9914, 9915, 9916, 9918, 9919, E9010, E9011, E9018, and E9019.^3^ The authors excluded hospitalizations of less than 18-year-olds and extracted demographic data such as age, sex, race, primary insurance payor, and hospital-level data including the region of the country where the hospital is located, hospital bed size for analysis. Race is defined by the HCUP and it was included as a variable because there are racial differences in the incidence, prevalence, and outcomes.^18^, ^19^, ^20^, ^21^ The Dayo modification of Charlson's comorbidity index was employed to further classify comorbid conditions. Outcomes of interest included invasive mechanical ventilation, hospital length of stay, in-hospital mortality, and cost of hospitalization.

### Statistical analyses

Continuous variables were summarized as means ± standard deviations or median (interquartile range), where appropriate. Categorical variables were summarized as counts (percentage of the study population). The authors compared baseline characteristics and in-hospital outcomes of patients with and without temperature-related illness using the Chi-Squared test for categorical variables and the Mann-Whitney *U* test for continuous variables. To assess for trends in the prevalence of temperature-related illness among AF hospitalizations by year, the authors performed a nonparametric test for trends across ordered groups using Cuzick's modification of the Wilcoxon rank-sum test, with “trend weights” for the database prior to the year 2011, as recommended. The predictors of in-hospital mortality were assessed using multivariable logistic regression. The authors included covariates with a significant effect on mortality in univariate analysis. Model specification and fit were assessed by the Wald Chi-Squared test and Akaike Information Criteria (AIC) respectively. All analyses were survey-weighted to account for the nature of the NIS data and performed at a two-tailed 5% level of significance using Stata version 16.1 (Stata Corporation, College Station, Texas).

### Ethics

This study did not require approval from our Institutional Review Board (IRB) because it uses a limited dataset, which is publicly available.

## Results

### Baseline characteristics

Out of a sample of over 38 million atrial fibrillation hospitalizations, the authors found a rather small yet noteworthy 37,933 encounters involving exposure to either heat or cold (Table 1). Among these, the median age was 79 years at the time of hospitalization, slightly older than the average of 78 years for non-TRI encounters. This included a larger share of patients both younger than 50 years (6.6% vs. 3%, *p <* 0.001) and older than 80 years (48.6% vs. 44.4%, *p <* 0.001) relative to the non-exposed group. Compared to females, males represented more TRI encounters (54.2% vs. 49.7%, *p <* 0.001). While Black patients represented 6.6% of the total AF encounters, they represented 12.2% of those with TRI. Whites, on the other hand, were 72% of the cohort and 67.7% of the TRI encounters. Thus, Blacks were overrepresented in the TRI group relative to the entire cohort.

**Table 1:** Baseline patient/hospital level characteristics and in-hospital outcomes of encounters analyzed.

Variable	No Exposure (No TRI) *n* = 38,246,148 (99.9%)	Exposure (TRI) *n* = 37,933 (0.1%)	p-value
Age, years (Median, IQR)	78 (69 – 85)	79 (67 – 86)	<0.001
Age Group, years			<0.001
18‒35	209,259 (0.5)	819 (2.2)	
36‒50	971,517 (2.5)	1601 (4.2)	
51‒65	5344,485 (14)	5673 (15)	
66‒80	14,726,080 (38.5)	11,420 (30.1)	
> 80	16,994,807 (44.4)	18,421 (48.6)	
*Sex*			<0.001
Male	19,016,867 (49.7)	20,570 (54.2)	
Female	19,228,400 (50.3)	17,364 (45.8)	
*Race*			<0.001
White	27,544,463 (83.2)	25,673 (77)	
Black	2531,293 (7.7)	4627 (13.9)	
Hispanic	1643,808 (5)	1505 (4.5)	
Asian or Pacific Islander	566,302 (1.7)	586 (1.8)	
Native American	133,455 (0.4)	202 (0.6)	
Other	667,250 (2)	733 (2.2)	
*Comorbidities*			
Hypertension	25,635,103 (67)	22,481 (59.3)	<0.001
Diabetes Mellitus	11,819,636 (30.9)	10,410 (27.4)	<0.001
Chronic Lung Disease	10,496,899 (27.4)	8161 (21.5)	<0.001
Congestive Heart Failure	8990,478 (23.5)	11,035 (29.1)	<0.001
Alcohol abuse	1011,180 (2.6)	3475 (9.2)	<0.001
Drug Abuse	368,114 (1)	1289 (3.4)	<0.001
Smoking	751,666 (19.7)	6850 (18.1)	<0.001
Dyslipidemia	14,464,395 (37.8)	9978 (26.3)	<0.001
Chronic Kidney Disease	8164,171 (21.3)	9021 (23.8)	<0.001
Chronic Liver Disease	788,893 (2.1)	1273 (3.4)	<0.001
Obesity	4085,247 (10.7)	2842 (7.5)	<0.001
Cancer +/- metastasis	2178,221 (5.7)	1673 (4.4)	<0.001
Thyroid Dysfunction	6680,140 (17.5)	6670 (17.6)	0.79
Obstructive sleep apnea	2431,259 (6.4)	1434 (3.8)	<0.001
*Charlson Comorbidity Index*			<0.001
0	6885,873 (18)	8644 (22.8)	
1	9479,326 (24.8)	9231 (24.3)	
2	8088,497 (21.1)	7155 (18.9)	
≥ 3	13,792,453 (36.1)	12,904 (34)	
CHA_2_DS_2_VASc Score			<0.001
0	1017,289 (2.7)	2545 (6.7)	
1	2852,675 (7.5)	3175 (8.4)	
2	5780,647 (15.1)	5668 (14.9)	
≥ 3	28,595,536 (74.8)	26,546 (70)	
*Hospital Region*			<0.001
Northeast	8144,899 (21.3)	6399 (16.9)	
Midwest	9514,418 (24.9)	10,456 (27.6)	
South	14,040,464 (36.6)	13,391 (35.3)	
West	6573,787 (17.2)	7687 (20.3)	
*Hospital Bed Size*			<0.001
Small	5463,046 (14.3)	5934 (15.7)	
Medium	9566,300 (25.1)	10,625 (28.2)	
Large	23,072,299 (60.6)	21,134 (56.1)	
*Hospital Location*			<0.001
Rural	4884,507 (12.8)	5730 (15.2)	
Urban, Nonteaching	15,870,123 (41.7)	14,653 (38.9)	
Urban, Teaching	17,347,014 (45.5)	17,309 (45.9)	
*In-hospital Outcomes*			
Invasive mechanical ventilation	1564,362 (4.1)	6251 (16.5)	<0.001
In-hospital mortality	1960,777 (5.1)	7061 (18.6)	<0.001
Length of stay, days [Median, (IQR)]	4 (2 – 7)	5 (2 – 8)	<0.001
Cost, US Dollars [Median, (IQR)]	8607 (4938–16,225)	10,082 (5574–19,144)	<0.001

Table 1 also shows that hospitalizations with TRI had a lower proportion involving obesity (7.5% vs. 10.7%), hypertension (59.3% vs. 67%), chronic lung disease (21.5% vs. 27.4%), smoking (18.1% vs. 19.7%), dyslipidemia (26.3% vs. 37.8%), cancer (4.4% vs. 5.7%), or obstructive sleep apnea (3.8% vs. 6.4%). These had a higher proportion presenting with congestive heart failure (29.1% vs. 23.5%), alcohol abuse (9.2% vs. 2.6%), drug abuse (3.4% vs. 1%), chronic kidney disease (23.8% vs. 21.3%), and chronic liver disease (3.4% vs. 2.1%). Overall, patient hospitalizations with TRI had fewer comorbidities than the non-TRI group (47.1% vs. 42.8% with one or no comorbidities). These encounters also featured a lower thromboembolic stroke risk (calculated using a CHA₂DS₂VASc score,^22^ as 15.1% vs. 10.2% reported either low (0) or moderate (1) risk. TRI encounters were overrepresented in the West (20.3% vs. 17.2%) and Midwest (28.2% vs. 25.1%) regions of the United States, and less represented in the Northeast (16.9% vs. 21.3%) region. Patients more frequently were hospitalized in small (15.7% vs. 14.3%) or medium (28.2% vs. 25.1%), and less so in large (56.1% vs. 60.6%) beds. A higher proportion of stays occurred in rural hospitals (15.2% vs. 12.8%) and fewer occurred in urban, non-teaching centers (38.9% vs. 41.7%). All the above values were significant.

### In-hospital outcomes

Table 1 likewise displays various in-hospital outcomes. These include a far higher proportion of invasive mechanical ventilation (16.5% vs. 4.1%, *p <* 0.001), in-hospital mortality (18.6% vs. 5.1%, *p <* 0.001), longer stays (5 [2‒8] vs. 4 [2‒7] days, *p <* 0.001), and bearing a $1475 higher median cost of treatment in US Dollars ($10,082 vs. $8607, *p <* 0.001) in TRI vs non-TRI hospitalizations respectively.

### Predictors of in-hospital mortality

**Fig. 1:**
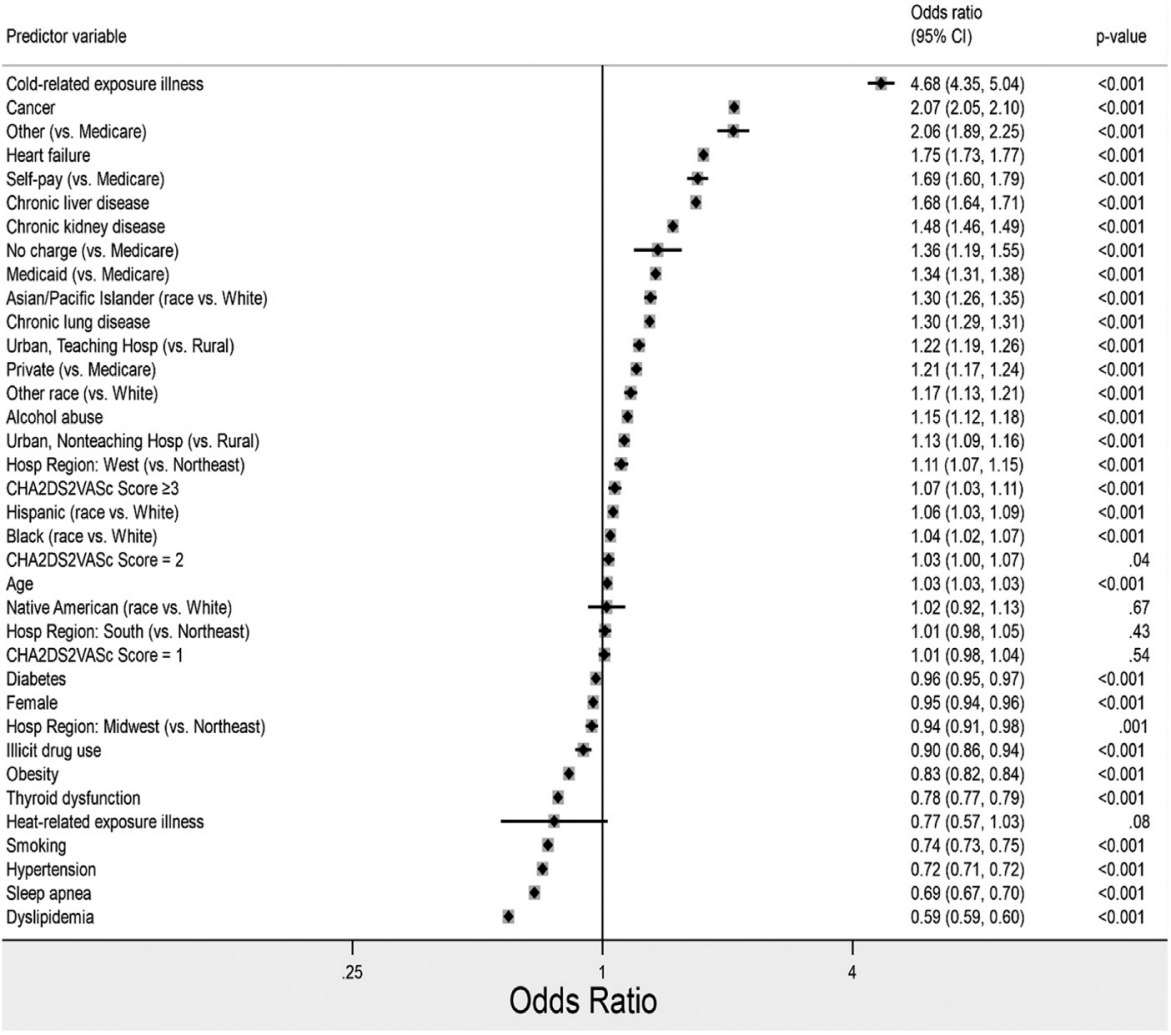
Multivariable Forest plots of the predictors of in-hospital mortality in atrial fibrillation encounters.

Factors predicting in-hospital mortality among AF hospitalizations were assessed using multivariable logistic regression and are shown as a forest plot in Fig. 1. All covariates were significant except for Native American race, heat-related exposure illness, CHA₂DS₂VASc score = 1, and hospital region: South. The single variable which showed by far the strongest predictor of in-hospital mortality was cold-related exposure illness at 4.68. Mortality odds were also markedly increased in those with cancer (2.07), Other insurance (vs. Medicare, 2.06), heart failure (1.75), self-pay (vs. Medicare, 1.69), chronic liver disease (1.68), chronic kidney disease (1.48), no charge (vs. Medicare, 1.36), Medicaid (vs. Medicare, 1.34), Asian/Pacific Islander ethnicity (vs. White, 1.30), chronic lung disease (1.30), stay at an urban teaching hospital (vs. rural, 1.22), private insurance (vs. Medicare, 1.21), “Other” race (vs. White, 1.16), alcohol abuse (1.15), stay at an urban nonteaching hospital (vs. rural, 1.13), and the West hospital region (1.11). Surprisingly, the odds of death were diminished in those with dyslipidemia (0.59), obstructive sleep apnea (0.69), hypertension (0.72), smoking (0.74), heat-related exposure illness (0.77), thyroid dysfunction (0.78), obesity (0.83), and history of illicit drug use (0.90). Several variables presented little effect on mortality odds, including age, gender, income, every race other than Asian/Pacific Islander, every hospital region other than West, and thromboembolic risk (via CHA₂DS₂VASc score).

### Trends in the prevalence of TRI in AF patients

**Fig. 2:**
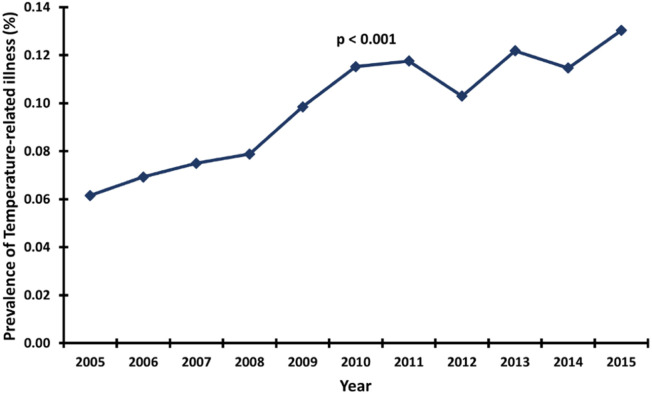
Linear trend in the prevalence of temperature-related illness among atrial fibrillation hospitalizations. p-value denotes a significant overall linear increase in the prevalence.

**Fig. 3:**
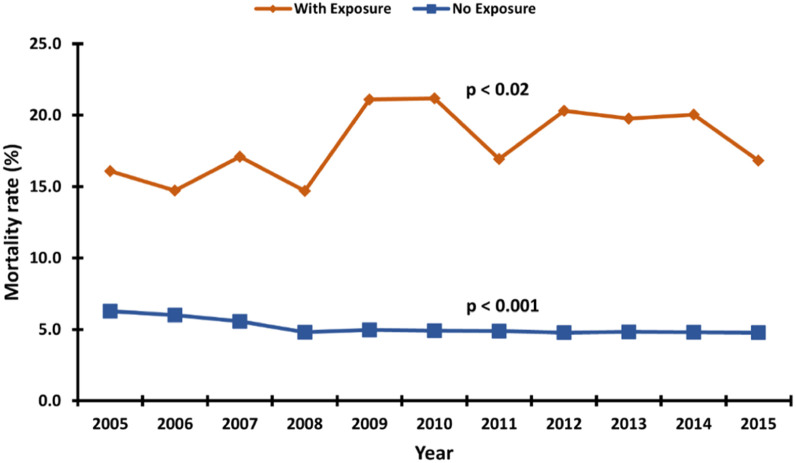
10-year trend in the mortality rate of atrial fibrillation encounters by temperature-related illness status. p-values show a significant linear trend in the mortality rate from 2005–2015.

**Fig. 4:**
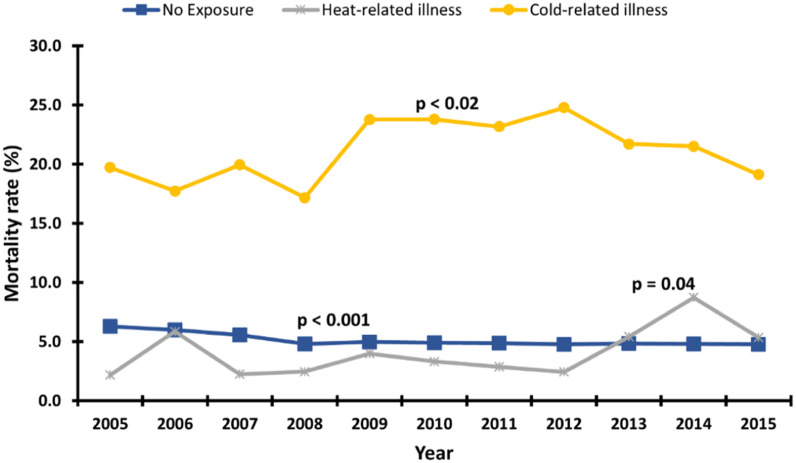
10-year trend in mortality rate of atrial fibrillation hospitalizations by form of temperature-related illness. p-values denote an overall trend towards linearity in the year-to-year changes in mortality rate for the groups.

Fig. 2 shows an increase in the prevalence of TRI from 2005 to 2015. In 2005, TRIs were associated with 0.06% of all atrial fibrillation hospitalizations, and by 2015 that number had more than doubled to 0.13%. Fig. 3 details a differential trend in the mortality rate of those atrial fibrillation encounters with and without TRI. While mortality in hospitalizations with and without TRI fluctuate at 14.7%–21.2% and 4.8%–6.3%, respectively, TRI-related hospitalizations grew 4% from 2005 to 2015 and non-TRI-related encounters declined 24% in that same period. An interesting trend emerges in Fig. 4. Cold-related illness forms a greater proportion of TRI occurrence among AF encounters who died. Our analysis estimates a cold-related mortality rate between 17.7%–19.1% (an 8% increase from 2005 to 2015), relative to a far smaller yet rapidly growing heat-related death rate from 2.2% in 2005 to 5.4% in 2015.

## Discussion

Exposure to non-optimal ambient temperatures, whether low temperatures or high represents a contribution to hospitalization as well as all-cause mortality.^23^ Both extremes have been shown to increase the risk of hospitalization and death due to cardiovascular and respiratory causes. Increases in mortality in patients with arrhythmias have been observed, particularly with respect to cold weather.^11^ In our sample, mortality in patients with a cold-weather illness was 4.68 times more likely (*p <* 0.001) than in patients without exposure, while in heat-related illnesses, patients were less likely to die (this result did not reach statistical significance). This suggests that in patients with atrial fibrillation, cold exposure presents a much greater threat than heat exposure. Importantly, atrial fibrillation patients living in colder geographical regions, especially during the winter may be at increased risk of death given the likely increased risk of experiencing cold weather TRI.

However, as other research has noted, indoor temperatures do not necessarily correlate to outdoor temperatures, and thus risk of experiencing TRI would change depending on the use of heating devices, equipment, or clothing and the frequency of exposure to outdoor low temperatures.^12^ In contrast to our results, previous reports have failed to show an association between changes in ambient temperature and the occurrence of arrhythmias^10^ or showed that heat increases mortality in patients with arrhythmias.^7^ The authors found a significant increase in the trends of TRI among AF hospitalizations, and our analysis also showed that cold-related injury was associated with increased odds of mortality in AF hospitalization. Our finding compares favorably with those of Nguyen et al. 2015,^12^ who showed an increased risk of ventricular arrhythmias in cold ambient temperatures.^9^ The strength of our study lies in the fact that the authors included only AF hospitalizations as opposed to a loosely defined arrhythmia group in the previous reports, which included other atrial arrhythmias and ventricular arrhythmias. Furthermore, our study considered mortality rates among hospitalized patients. These differences likely contribute to differences in findings. There is a significant increase in the prevalence of TRI within atrial fibrillation hospitalizations from 2005 to 2015. Among TRI patients, there was a higher proportion of hospital mortality, length of stay, and mechanical ventilation. These factors, among others, likely contributed to the overall increase in the cost of hospitalization in TRI encounters.

The authors further evaluated the prevalence of TRI and mortality risk in various sub-groups to aid in the identification of high-risk patients. All races had significantly higher odds of death compared to Whites except for Native Americans (Fig. 1). There were fewer TRI encounters in the Northeast of the country compared to the other regions. When examining the odds of mortality in this sub-group, the authors saw increased odds in the West, and decreased odds in the Midwest region, compared to the Northeast (Fig. 1). Generally, the West and South regions of the United States tend to have less frequent cold weather events,^24^ and residents and local governments may be less equipped to deal with excessive cold weather, thus residents in these regions may be more likely to develop cold-related injuries. Among hospital location sub-groups, patients in rural areas were the least represented in the TRI group. They also had lower odds of mortality compared to the two urban subgroups (Fig. 1). Non-optimal temperatures may present a risk for the occurrence of AF. Cold temperature is associated with increased vagal tone, with a resultant decrease in baseline heart rate, which is associated with an increased risk of developing AF.^25^^,^^26^ These differences may be due to a higher proportion of heat-related TRI in rural populations, differences in TRI severity between groups different behavioral responses (i.e., better winter preparedness/attention to weather alerts) to extreme weather regionally. However, the NIS database does not contain data elements to allow a further investigation of this suspicion.

The following comorbidities were found in lower proportions in the exposure group: obesity, hypertension, chronic lung disease, smoking, dyslipidemia, cancer, and obstructive sleep apnea. All these except cancer and chronic lung disease also had lower odds of mortality in our analysis. Congestive heart failure, alcohol abuse, drug abuse, chronic kidney disease, and chronic liver disease all had higher proportions in the exposure group. All of these except for drug abuse were associated with higher mortality risk. These groups may be at higher risk of TRI itself, atrial fibrillation itself, or are more susceptible to atrial fibrillation associated with TRI. White race, female sex, and younger age were all associated with reduced odds of mortality. There was a higher incidence of TRI among males compared to females (54.2% vs. 45.8%) and female sex was associated with lower odds of mortality in patients with TRI. This difference may be due in part to the effect of estrogen neuronal thermoregulation in extremes of temperature. Sex differences exist in the initiation of neurohormonal thermoregulation in mouse models of energy deficiency.^27^

AF is the most common sustained arrhythmia worldwide. Given our finding of the increasing trend in prevalence, there is a need to rapidly identify individuals with arrhythmias such as AF who are at risk for increased adverse outcomes based on forecasted changes in environmental temperatures. When combined with patient demographics as well as local or regional information on health care outcomes, the presence of TRI may present an increased risk of mortality in the general population. Thus, the presence of TRI, especially cold-related injury in AF patients presenting to the emergency department should prompt vigilant monitoring and careful interventions to mitigate the risk of potential excess mortality. The rising cost of healthcare expenditure in the United States presents a challenge to healthcare systems and all levels of government. This may lead not only to improvement in patient-level outcomes but also potentially a reduction in the associated cost of hospitalization in these patients.

Limitations to our study exist which should drive further research, many of which are limitations in the NIS dataset used. These include but are not limited to, the inability to validate diagnoses, determine the severity of TRIs, determine information regarding arrhythmia onset, and determine causes of death. The authors also could not examine specific causes of death within our cohort, only all-cause mortality risk. Since the NIS database is a hospital administrative database, it is subject to in-hospital level coding bias, where out-of-hospital event rates are not captured. This may lead to underestimating true mortality rates and limit the generalizability of our findings to the community. Studies involving TRI subgroups are needed to drive decision-making and risk evaluation both regionally and seasonally while studies that assess specific causes of mortality, and the severity of TRI are needed to inform potential treatment decisions. Further, the authors suspect that arrhythmias may enhance the deleterious effect of TRI. However, owing to logistic limitations, the authors were unable to compare TRI occurrence between AF and non-AF groups. Follow-up studies are needed to clarify the relationship between AF and TRI. The authors elected to remove observations with missing data from our models, which may have reduced the statistical power of our analysis and introduced biases in our estimates. Finally, while the authors controlled for many confounding variables via multivariate analysis, it is possible that other factors exist to confound our results such as socioeconomic status, homelessness status, or association with specific adverse weather events such as heatwaves or cold fronts. The authors cannot draw conclusions regarding physiologic reasons for our results, but other studies suggest that increased sympathetic responses under cold weather stress could be driving an increased risk of arrhythmia.^9^ Our results could be due to a marked increase in sympathetic responses due to cold weather itself and to cold weather TRI, however, more research is required to come to this conclusion.

## Conclusion

The occurrence of TRI among hospitalized AF patients is small, but there is an increasing trend in the prevalence, which more than doubled over the decade reviewed in this study. Individuals with AF who are admitted with a TRI face significantly poorer outcomes, including increased mortality, as compared to those admitted without a TRI. Public health interventions, such as regular announcements to educate the public on the dangers of TRI, may increase awareness and offer practical steps to limit prolonged exposure to the elements. In addition, targeted interventions aimed at patients with chronic illnesses, such as AF and congestive heart failure, may help improve outcomes among at-risk patients. Further research should be performed to elucidate the relationship between increasing age, social inequality, duration of AF, and other comorbidities – including congestive heart failure and diabetes mellitus – and TRI. An improved understanding of the complex interplay between cardiovascular pathology and TRI may play an important role in developing effective strategies for prevention, management, and monitoring.

## CRediT authorship contribution statement

**Daniel Antwi-Amoabeng:** Conceptualization, Formal analysis, Writing – original draft, Writing – review & editing. **Sunil Sathappan:** Writing – original draft. **Tarek R. Firzli:** Writing – original draft. **Bryce D. Beutler:** Writing – original draft, Writing – review & editing. **Mark B. Ulanja:** Writing – original draft, Writing – review & editing. **T. David Gbadebo:** Supervision, Writing – review & editing.

## Declaration of Competing Interest

The authors declare that they have no known competing financial interests or personal relationships that could have appeared to influence the work reported in this paper.
